# Prevalence of acute olfactory dysfunction differs between variants of SARS-CoV-2—results from chemosensitive testing in wild type, VOC alpha (B.1.1.7) and VOC delta (B.1617.2)

**DOI:** 10.1007/s00405-022-07431-6

**Published:** 2022-06-29

**Authors:** Constantin A. Hintschich, Veronika Vielsmeier, Christopher Bohr, Jan Hagemann, Ludger Klimek

**Affiliations:** 1 Department of Otorhinolaryngology, Regensburg University Hospital, Franz-Josef-Strauss-Allee 11, 93053 Regensburg, Germany; 2 Department of Otorhinolaryngology, Mainz University Hospital, Mainz, Germany; 3 Center for Rhinology and Allergology, Wiesbaden, Germany

**Keywords:** SARS-CoV-2, COVID-19, Olfaction, Smell

## Abstract

**Background:**

Olfactory dysfunction is one of the leading symptoms of COVID-19. Previous data suggest a different prevalence between the wild type virus and its subsequent variants. Here, we report on a prospective study to psychophysically compare olfactory function in acute SARS-CoV-2 infection between wild type, VOC alpha and VOC delta.

**Methods:**

SARS-CoV-2 was confirmed by reverse-transcription quantitative real-time PCR and virus variants were differentiated by high-sensitive next-generation sequencing. Home-quarantined were sent a validated and blinded smell identification test. A detailed instruction ensured correct self-administration.

**Results:**

A total of 125 patients were included in study. Patients with the wild type of SARS-CoV-2 self-evaluated their olfactory function significant lower on the visual analog score compared patients with the VOCs alpha or delta (4.1 ± 1.5 vs. 6.8 ± 2.9 and 7.3 ± 0.9; *p* < 0.001). Likewise, a significant difference of the prevalence of psychophysically confirmed hyposmia (wild type: 73%; alpha: 41%; delta 48%; *p* < 0.01) and smell test score (48 ± 25% vs. 70 ± 23% and 67 ± 18%; *p* < 0.01) could be seen between wild type on one side and VOCs alpha and delta on the other side.

**Conclusion:**

In this study, both self-reports and psychophysical testing revealed a significant higher prevalence of olfactory impairment in the wild type of SARS-CoV-2 compared to the VOCs alpha and delta.

## Introduction

Like other organisms, viruses are also subject of evolution: The coding RNA constantly mutates, which may change also viral properties. Single or cumulated mutations may potentially result in an evolutionary process and lead to the domination of superior novel virus strains. Also, for SARS-CoV-2 virus variants have changed the course of the pandemic and might even endanger a global immunity through an increased contagiosity and immune escape:

In central Europe, B.1.1.7 (variant of concern (VOC) alpha) took over the dominance of the so-called wild type in the beginning of 2021 but was then quickly displaced by the even more contagious B.1.1617.2 (VOC delta). B.1.1.529 (VOC omicron) was only discovered in late November 2021 and has already spread worldwide.

While the epidemiological and immunological properties of the single VOCs have been investigated in detail the difference in its clinical symptoms is less known [1, 2]. Although, a lower prevalence of chemosensitive dysfunctions have been described anecdotally, only very few scientific publications compared the wild type and its VOC successors [3, 4].

Here, we report on a chemosensitivity study comparing for the first time the severity and prevalence of olfactory dysfunction in acute SARS-CoV-2 infection between the wild type, and the VOCs alpha and delta.

## Methods

This multicenter prospective study was conducted between April 2020 and April 2021 at the Department of Otorhinolaryngology of Regensburg University Hospital and the Department of Otorhinolaryngology of Mainz University Hospital in cooperation with the Center for Rhinology and Allergology in Wiesbaden, Germany. It was conducted after ethics committee approval (University Regensburg approval number 20-1766_6-101; Landesärztekammer Rheinland-Pfalz approval number 14943) and performed in accordance with the Declaration of Helsinki and its later amendments. Detailed information was provided to the patients in written form and their consent was obtained in written form, too.

SARS-CoV-2 infection was confirmed by reverse-transcription quantitative real-time PCR (RT-qPCR). After the emergence of novel virus variants, high-sensitive next-generation sequencing differentiated between the wild type, B.1.1.7 and B.1.617. Home-quarantined patients were then contacted and sent a validated and blinded smell identification test (8-item NHANES Pocket Smell Test, Sensonics, Haddon Heights, NJ, US or 16-item Identification test, Burghart Messtechnik, Holm, Germany). A detailed instruction ensured correct self-administration in home quarantine. Normosmia was defined as ≥ 75% correct answers, respectively.

Data were analyzed using SPSS Statistics software (version 26, IBM, Armonk, NY, USA). Graphs were illustrated using Prism software (version 9, GraphPad Software, San Diego, CA, USA). Values are expressed as mean ± standard deviation (SD), and *p* < 0.05 was considered statistically significant. Data were tested for statistical significance using one-way ANOVA and Tukey’s post hoc multiple comparison test.

## Results

A total of 125 patients (mean age 41 ± 16 years) were included in this study; 55 (44%) were female, and 70 (56%) were male. 63, 41, and 21 patients were diagnosed with wild type (between April 2020 and April 2021), VOC alpha (between February and April 2021), and VOC delta (between June and December 2021), respectively.

At the timepoint of the psychophysical testing patients self-rated their olfactory function as 5.8 ± 4.2 on the visual analog score (VAS). However, we found a substantial difference when comparing the three strains: Patients who were diagnosed with the wild type of SARS-CoV-2 self-evaluated their olfaction as 4.1 ± 1.5 on the VAS. In contrast, patients with the alpha or delta variant self-estimated their olfactory function significantly better compared to the wild type cohort (VAS 6.8 ± 2.9 and 7.3 ± 0.9, respectively; *p* < 0.001).

**Fig. 1:**
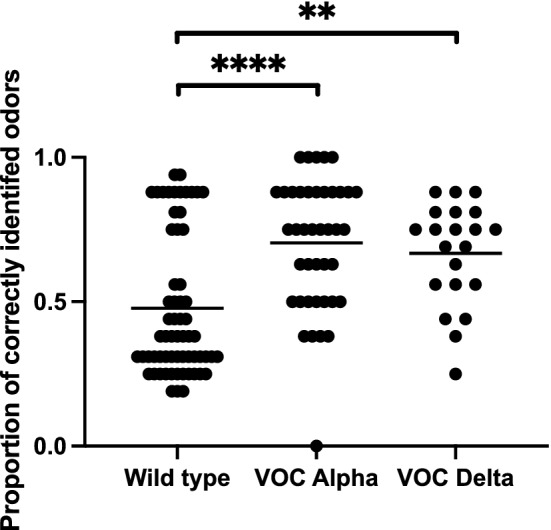
Proportion of correctly identified odors for wild type, VOC alpha, and VOC delta; **p < 0.01, ****p < 0.0001

Interestingly, this difference between wild type and both alpha and delta variant was confirmed through psychophysical testing: Patients with wild type were tested significantly more often hyposmic than patients with the alpha or delta virus variants, (wild type: 73%; alpha: 41%; delta 48%; *p* < 0.01). Also, the test score of patients with wild type was significantly lower than in patients with the alpha or delta variant, respectively (48 ± 25% vs. 70 ± 23% and 67 ± 18%, respectively; *p* < 0.01; Fig. 1).

## Discussion

To the best of our knowledge, this is the first study of its kind comparing psychophysically assessed olfactory function between wild type SARS-CoV-2 and the VOCs alpha and delta.

Patients with the alpha or delta variant evaluated their subjective smell significantly better compared to the wild type cohort. This is in line with previous publications for the alpha variant [3, 4] and an anecdotal report for the delta variant.

In addition to patients’ subjective ratings, we assessed olfaction using validated psychophysical tests: Likewise, a significant difference for both prevalence and test score could be seen between wild type on one side and VOCs alpha and delta on the other side (Fig. 1).

Interestingly, between the VOCs alpha and delta no significant difference could be detected in either subjectively or psychophysically assessed olfaction. Similar findings which were only based on patients self-estimation have been reported previously in a preprint [5].

Limitations of this study are the small cohort size and the low age of the delta cohort compared to both the wild type cohort and the alpha cohort (wild type: 43 ± 16 years; alpha: 43 ± 17 years; delta 31 ± 8 years; *p* < 0.05).

The underlying mechanisms of interstrain differences in hyposmia are still elusive. A previous meta-analysis associated the mutation D614G with a higher prevalence of hyposmia [6]. However, as both lineages VOC alpha and VOC delta express D614G [7], this cannot explain their decreased olfactory dysfunction in comparison to the wild type virus. Infections and vaccinations lead to an increasing immunity during the course of the pandemic. Hence an improved immunity might have led to a lower prevalence of olfactory disorders in later virus strains such as VOC alpha or VOC delta. Contrary, in upper respiratory tract infections due to other viruses than SARS-CoV-2 olfactory dysfunction is relatively frequent [8]. Thus, further studies are necessary to identify the exact pathomechanisms of olfactory impairment in COVID-19 and to compare its virus mutations with other seasonal cold viruses.
